# Evaluation of Anthocyanin Profile, Antioxidant, Cytoprotective, and Anti-Angiogenic Properties of *Callistemon citrinus* Flowers

**DOI:** 10.3390/plants9081045

**Published:** 2020-08-17

**Authors:** Giuseppina Laganà, Davide Barreca, Antonella Smeriglio, Maria Paola Germanò, Valeria D’Angelo, Antonella Calderaro, Ersilia Bellocco, Domenico Trombetta

**Affiliations:** Department of Chemical, Biological, Pharmaceutical and Environmental Sciences, University of Messina, Via G. Palatucci, 98168 Messina, Italy; laganag@unime.it (G.L.); asmeriglio@unime.it (A.S.); germanom@unime.it (M.P.G.); vdangelo@unime.it (V.D.); anto.calderaro@gmail.com (A.C.); ebellocco@unime.it (E.B.); dtrombetta@unime.it (D.T.)

**Keywords:** *Callistemon citrinus* (Curtis) Skeels, anthocyanins, antioxidants, cytoprotection, anti-angiogenic activity

## Abstract

Lemon bottlebrush (*Callistemon citrinus* (Curtis) Skeels) is one of the most common ornamental plants, diffused worldwide, and characterized by the presence of flowers with an intense red/purple coloration. There is increasing interest in the use and application of anthocyanins for their unique structural/chemical features in both food and pharmaceutical applications. RP-HPLC-DAD-ESI-MS/MS analysis of an enriched fraction of acidified methanolic extract of *C. citrinus* flowers allow the possibility of identifying, for the first time, the presence of four anthocyanins: cyanidin-3,5-*O*-diglucoside (cyanin), peonidin-3,5-*O*-diglucoside (peonin), cyanidin-3-*O*-glucoside, and cyanidin-coumaroylglucoside-pyruvic acid. Moreover, the evaluation of antioxidant and biological potential showed a remarkable activity of this fraction, able to actively scavenge DPPH, AAPH, and ABTS radicals, and to counteract the β-carotene-bleaching. In addition, it protects human mononuclear cells from oxidative injuries and prevents angiogenesis (acting in the range of few μg/ml); furthermore, it does not show significant iron-chelating ability (up to 200 µg/mL). The easy way of cultivation, robustness, and adaptability to different environments make the flowers of this plant a useful source of anthocyanins, with remarkable health promoting properties.

## 1. Introduction

Lemon bottlebrush (*Callistemon citrinus* (Curtis) Skeels) is a shrub that is native to the Australian continent, belonging to the Myrtaceae family, where it grows wild. Nowadays, it is diffused worldwide as an ornamental garden plant, due to its hardness and adaptability. Moreover, it is characterized by striking red flower spikes with a lemony scent (hence, the common name), which is present for almost all of the months of the year on the plant, but mainly in November and December [1,2,3,4,5]. It is commonly known as crimson bottlebrush, red bottlebrush, or lemon bottlebrush. It grows up to 4–5 m tall, but commonly reaches 1–3 m. The flowers, in particular, are characterized by a red color, and they are arranged in spikes (with a diameter of 45–70 mm and a length up to 60–100 mm) on the ends of branches that continue to grow after flowering (and, sometimes, also in the upper leaf axils). In each flower, there are about 30–50 stamens of red color and the anthers of purple color.

In addition to being an ornamental plant, *C. citrinus* is used for its therapeutic potential in ethno-pharmacology [6,7,8,9]. Lemon bottlebrush is utilized in folk medicine to treat several disorders, such as hemorrhoids, dysentery, rheumatism, tuberculosis, bronchitis, urinary incontinence, excessive menstruation, or mucosal discharge [6,7,8,9,10,11,12]. Recently, Fayemi et al. analyzed the bioactivities of phytochemicals in *C. citrinus* against multi-resistant food borne pathogens, α-glucosidase, and MCF-7 cancer cell line, with promising results [13]. Antimicrobial activity of lyophilized extracts from flowers and leaves of *C. citrinus* has also been reported against *Listeria monocytogenes* in beef burgers [14]. The phytochemical screening showed the presence of polyphenols, alkaloids, monoterpenoids, aliphatic acids, tannins, sesquiterpenes, triterpenoids, and steroids in different parts of the plant (such as leaves, stem backs, flowers, and seeds [15,16,17,18,19,20,21,22,23,24]), showing the potential of this shrub for the development of products with health promoting properties. Although some scientific information on this plant is present in literature [15,16,17,18,19,20,21,22,23,24], to date, no studies are available about the anthocyanin profile of the flowers. Anthocyanins are the glycosylated forms of anthocyanidins responsible for the colors (red, purple, and blue) in fruits and vegetables, and some health promoting foods (such as berries, currants, grapes, and some tropical fruits) show high anthocyanin content [25,26,27,28,29,30]. Moreover, these compounds have remarkable biological and pharmaceutical potential, and several scientific reports on cell cultures, animal models, human clinical trials, and epidemiological studies showed their strong health promoting properties [31,32,33,34,35,36,37]. Based on these observations, the aim of this study is to shed some light on the anthocyanin profile and to evaluate the antioxidant, cytoprotective, and anti-angiogenic properties of the enriched fraction of acidified methanolic extract of *C. citrinus* flowers (EAC).

## 2. Results

### 2.1. Identification and Quantification of Anthocyanins by Reverse Phase- High Performance Liquid Chromatography Coupled with Diode Array and Electrospray Mass Spectrometry Detection (RP-HPLC-DAD-ESI-MS/MS) 

**Figure 1:**
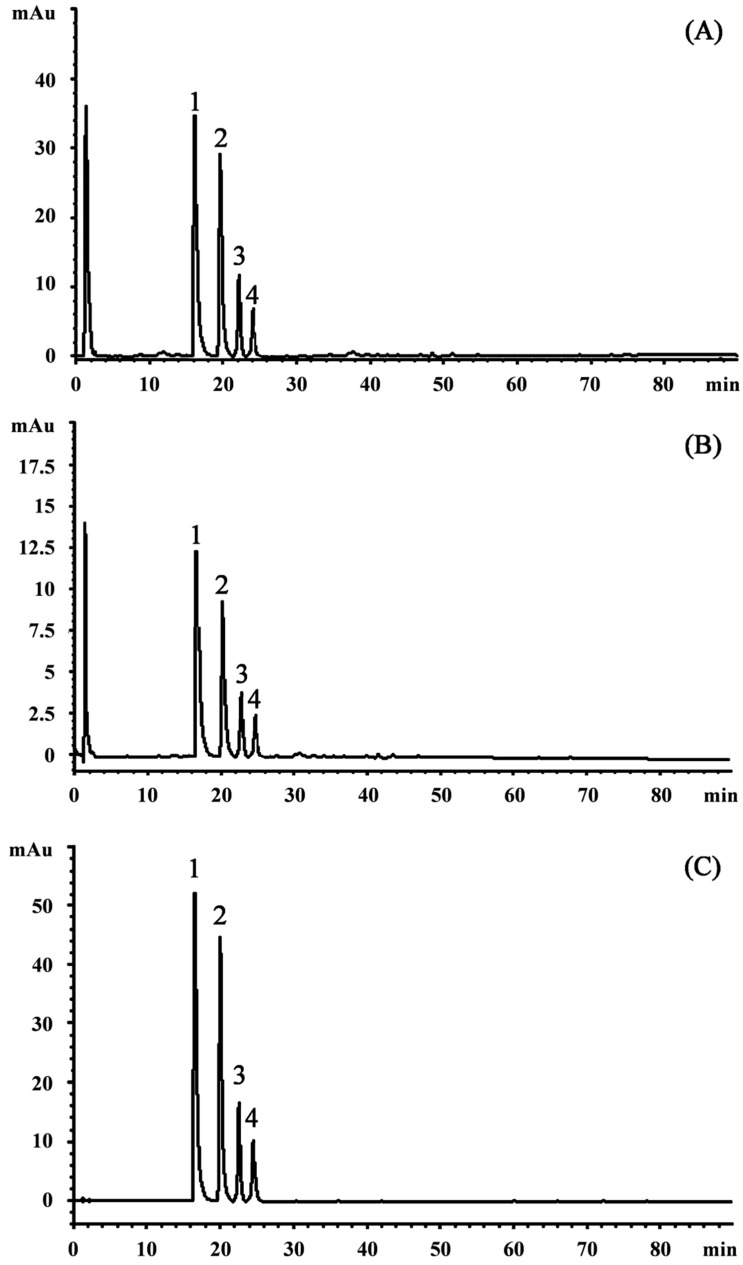
Representative RP-HPLC-DAD-ESI-MS/MS chromatogram of enriched fraction of acidified methanolic extract of *C. citrinus* flowers (EAC) acquired at 292 nm (**A**), 330 nm (**B**), and 520 (**C**) nm. **1** cyanidin-3,5-*O*-diglucoside (cyanin), **2** peonidin-3,5-*O*-diglucoside (peonin), **3** cyanidin-3-*O*-glucoside, **4** cyanidin-coumaroylglucoside-pyruvic acid.

Anthocyanins, either in dietary or non-dietary forms, are very interesting compounds with promising health properties. Being natural antioxidants, they may decrease oxidative stress, restoring the physiological antioxidant status [25,29,38]. Moreover, they may improve the quality and shelf life of several products by preventing oxidative rancidity and browning reactions in fat-based foods, meat, and dairy products [29]. This is the first manuscript focusing on a detailed RP-HPLC-DAD-ESI-MS/MS separation and identification of anthocyanin’s profile of EAC (Figure 1). The analysis showed an interesting polyphenol profile characterized by the predominance of anthocyanins. Indeed, it is possible to observe from Figure 1, which shows the HPLC-DAD chromatogram acquired at 292 (A), 330 (B), and 520 (C) nm that no compound belonging to the other polyphenol class has been detected in significant amount, with respect to anthocyanins.

The inspection of UV-visible spectra, the MS, and MS/MS fragmentation patterns, let us identify four compounds belonging to anthocyanins. The four anthocyanins were identified and quantified (Table 1) as cyanidin-3-*O*-glucoside (CyG) equivalents, of which the most abundant one is cyanidin-3,5-*O*-diglucoside (295.58 ± 2.226 mg CyG/100 g of dry extract (DE), followed by peonidin-3,5-*O*-diglucoside, cyanidin-3-*O*-glucoside, and a more complex cyanidin-derivative (cyanidin-coumaroylglucoside-pyruvic acid). Poli-glycosylated and polymeric anthocyanins, in particular cyanidin derivatives, are very frequently found in red flowers, as observed previously [38]. In addition, the presence of cyanidin-3,5-*O*-diglucoside was already found in flowers of another Callistemon species, *Callistemon viminalis* [39].

### 2.2. Antioxidant Activities

**Figure 2:**
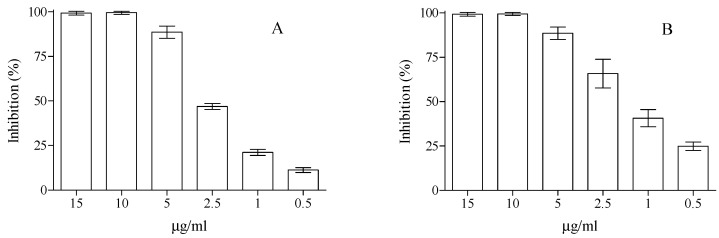
Radical scavenging activity of EAC measured by (**A**) DPPH and (**B**) TEAC assays. Data represent mean ± SD (*n* = 3) and were expressed as inhibition %.

In order to establish the antioxidant potential of the EAC, we have performed the most common antioxidant assays, such as 2,2-diphenyl-1-picrylhydrazyl (DPPH), trolox equivalent antioxidant capacity (TEAC), ferric ion reducing antioxidant power (FRAP) and oxygen radical absorbance capacity (ORAC) assays, β-carotene bleaching, and iron chelating capacity. As can be seen in Figure 2, EAC showed a well-defined dose-dependent antioxidant and free-radical scavenging activity towards DPPH^•^ and ABTS^•+^, with a remarkable activity, just at very low concentration 1–2.5 µg/mL. Indeed, for instance, 2.5 µg/mL has been able to scavenge ~47 and 66% of radical species in the DPPH and TEAC antioxidant assays, respectively. The obtained half-maximal inhibitory concentration (IC_50_) values were 2.2 (C.L. 95% 1.829–2.775) and 1.64 (C.L. 95% 1.331–2.133) µg/mL for DPPH and TEAC antioxidant assays, respectively. Moreover, in the ferric ion reducing antioxidant power (FRAP), where the reduction of ferric iron to ferrous iron in the presence of the molecules with antioxidant power is monitored, EAC showed remarkable potentiality with a FRAP value of 73.63 ± 3.53 μM trolox equivalents (TE). The extract under study also showed strong free-radical scavenging activity in β-carotene and ORAC assays, with an IC_50_ of 5.66 μg/ml (C.L. 95% 4.450–7.210) and 0.73 μg/mL (C.L. 95% 0.622–0.854), respectively. Due to the great importance of chelating processes of metal in the biological environment, we have tested the potential iron-chelating ability of the extract in the ferrozine assay. Although the extract has supplied interesting results in the above-described assays, in this last one, it did not have significant biological potential, up to the final concentration of 200 µg/mL (data not shown).

### 2.3. Cytoprotective Activity

**Figure 3:**
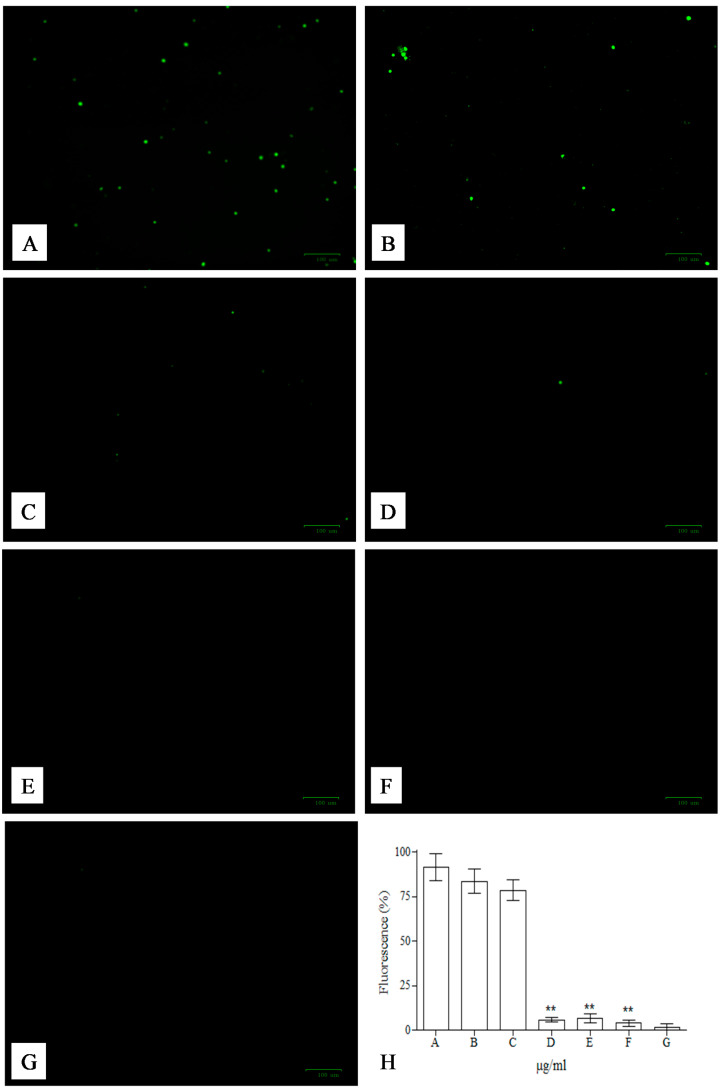
Intracellular reactive oxygen species (ROS) detection on human mononuclear cells t-BOOH treated with DCFH-DA. Mononuclear cells were treated with 50 μM t-BOOH (**A**) in the absence or in the presence of 2.5 (**B**), 5 (**C**), 10 (**D**), 25 (**E**), and 50 (**F**) μg/mL of EAC. Cells incubated in the same condition, but without t-BOOH and extract (**G**), were used as positive control. The green fluorescence, characteristic of DCF, was measured using ZOE Fluorescent Cell Imager. The elaboration of the fluorescence of the image has been performed with Image J software and the obtained arbitrary unit of fluorescence expressed in percentage (**H**). Data shown are representative of three independent experiments. Asterisks (**) indicate a significant difference with respect to control (*P* < 0.05).

The preliminary tests (performed for the evaluation of potential cytotoxic effects of the compounds present in the EAC) did not reveal significant changes in the vitality of mononuclear cells (monitored by trypan blue coloration) in the range of concentration of 0.0–100.0 µg/mL (data not shown), revealing good tolerability of the compounds present in EAC. Above these concentrations (125, 150, 175, 200 µg/mL), concomitantly with the increase of concentrations, the mortality reached ~50% at 200 μg/mL, after incubation for 24 h at 37 °C (data not shown). Once again, in this assay arises the double behavior of secondary metabolites, which over-fixed concentrations start to show pro-oxidant (rather than antioxidant) activity, as already reported for other flavonoids [40,41,42,43,44,45]. One of the first evidences of both activities of these compounds has been reported by Abuja et al. [45]. They tested an extract of elderberry (rich in anthocyanins), which showed both antioxidant and pro-oxidant activities, as far as the oxidation of low-density lipoproteins are concerned. These double activities are due to the time of the addition of the extract and to the presence of copper. In fact, these compounds are molecules with several biological potentials and the same properties that make these compounds useful for biological functions (often at low concentrations), change the characteristics of the same compounds at higher concentrations, making them potentially dangerous. The presence of transition metals can shift behavior of anthocyanins from antioxidants to pro-oxidants, increasing reactive oxygen species (ROS) generation, and damage biological macromolecules, such as proteins and DNA. In particular, the presence of a catechol B-ring in anthocyanins basic skeleton (as in the case of cyanidin and its derivatives) has lower reduction potentials and strong pro-oxidant activities [40]. The pro-oxidant activity of anthocyanins can be also due to inhibition of catalase (antioxidant enzyme that decomposes hydrogen peroxide) and, of consequence, to an indirect increase of oxidative stress and onset of apoptosis, as shown by Scheit and Bauer [42]. Taking into account the concentrations below 100.0 µg/mL, the potential of EAC to fight strong oxidants, such as t-BOOH, has been analyzed on monocellular cells, and detected with 2,7-dichlorodihydrofluorescein (DCFH), the most widely used fluorescent probe for the detection of intracellular ROS. The 2,7-dichlorodihydrofluoresceindiacetate is a freely diffusible, non-fluorescent compound that crosses cell membranes, and it is hydrolyzed by intracellular esterases and retained intracellularly to form the active probe. It is commonly used to monitor intracellular oxidants produced by oxidative stress or apoptosis, based on one-electron oxidation process that yields a radical intermediate, which is, afterward, oxidized to form highly resonant moieties responsible for the increase of the fluorescence probe value. The incubation of the cells with t-BOOH results in a clear increase in the fluorescence (Figure 3A), but the presence of 10–50 μg/mL (Figure 3B–D) of EAC resulted in the complete neutralization of the activity of the strong oxidant, while at low concentrations 2.5–5 μg/mL (Figure 3E,F), the effects were almost completely negligible. The elaboration of the arbitrary units of fluorescence of sample images and statistical analysis are in agreement with the above-described observation, with values almost completely superimposable with those obtained in the control sample for the concentration ranging from 10–50 μg/mL (Figure 3H). Basal ROS levels were unaffected by the exposure to the utilized concentrations of EAC (data not shown).

### 2.4. Anti-Angiogenic Effect

**Figure 4:**
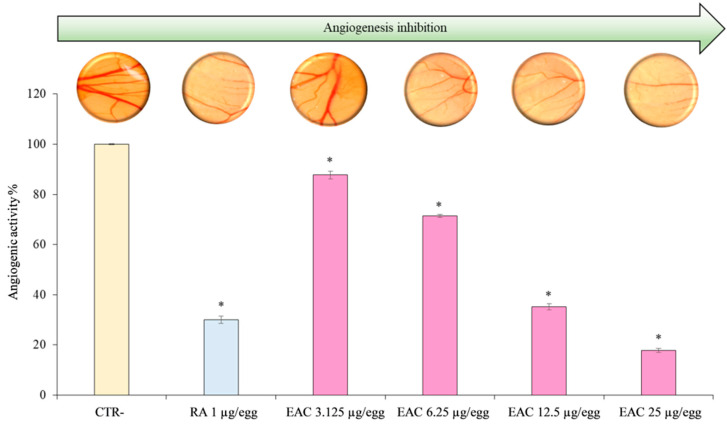
Results were expressed as angiogenic activity percentage (%) of different concentrations of EAC with respect to negative control (CTL−) and retinoic acid (RA) 1 μg/egg (CTL+) and as representative microscopic images of the vascular networks after treatment; * *P* < 0.001 vs. CTL−.

Among the several in vivo assays, which have been developed over time to evaluate the angiogenesis, the chick chorioallantoic membrane (CAM) assay represents the most simple, quick, and inexpensive model. The anti-angiogenic effect of the extract, under study, evaluated in a concentration range, which did not affect the cell viability, was reported in Figure 4. The EAC was able to inhibit in a dose-dependent and statistical significant manner (*P* < 0.001) CAM vessel growth in the concentration range 3.125–25.0 μg/egg from 12.29 to 89.11%, with respect to negative control (Figure 4) showing a very interesting IC_50_ value (9.80 μg/egg, C.L. 95% 8.403–11.421). Retinoic acid, used as a positive control (1.0 μg/egg) for its well-known angiostatic power, exhibited an anti-angiogenic activity of 69.98%. Representative microscopic images of the CAM vascular networks for each treatment were reported on corresponding graph bar (Figure 4). Images showed clearly that the number, length, and size of the junction vessels were decreased after the CAM treatment with the different EAC concentrations in comparison with the negative control. Moreover, results comparable or even higher in terms of thinner vascular networks (Figure 4) have been observed with the EAC highest concentrations 12.5 and 25.0 μg/egg (64.81% and 82.11%, respectively), with respect to retinoic acid (1 μg/egg) as positive control (69.98%). 

## 3. Discussion

Recently, anthocyanins have been gaining increasing attention due to their unquestionable health promoting properties and to the possibility to modify the basic skeleton with several functional groups, able to increase different biological and pharmaceutical applications of this molecules.

The mechanisms that underline the nutraceuticals potentials of anthocyanins are complex and involve the modulation of several biochemical pathways (including the activation of free-radical scavenging, mitogen-activated protein kinase, cyclooxygenase, and inflammatory cytokine signaling pathways), and the modulation of cell cycle and membranes. RP-HPLC-DAD-ESI-MS/MS analysis let us to identify, for the first time, the presence of four major anthocyanins in the EAC. Cyanidin-3,5-*O*-diglucoside (cyanin) and peonidin-3,5-*O*-diglucoside (peonin) are, by far, the most abundant derivatives, with values ranging in the ~180–300 mg/100 g of DE, while the other two derivatives are present in the range of 13–38 mg/100 g DE. The identification of these compounds is interesting because the two major compounds is present in remarkable amounts, supporting our hypothesis to utilize the flower of *C. citrinus,* due to the robustness and adaptability of the shrub to different soil and environmental conditions, as a source of these interesting nutraceutical compounds for their utilization in different industrial applications. The analysis of antioxidant, cytoprotective, and anti-angiogenic properties of this EAC further support these potentials. Our metabolism and lifestyles, which expose organisms to many different stresses, generate, daily, a remarkable amount of reactive oxygen species (ROS) involved in illness conditions that can bring to the development of pathological states, influencing human wellness. Proanthocyanidins, and even more, anthocyanins, are one of the most promising and strong antioxidants among the flavonoid compounds [25,29,46,47,48,49,50,51]. This behavior has been confirmed by the data obtained in free radical scavenging assays performed in this study, based on both hydrogen atom transfer and electron transfer-based methods. 

In the former, the EAC shows high values of IC_50_ in the ORAC and in the β-carotene bleaching assay, highlighting the capacity of the compounds present in the enriched fraction to perform a reaction scheme based on competition of antioxidant and substrate for the generation of peroxyl radicals through the decomposition of azo-compounds. Moreover, in the assays based on electron transfer-based methods (DPPH, FRAP, TEAC), the EAC shows remarkable value of IC_50_, indicating the presence of compounds able to perform the reduction of the oxidants present in the solution. Among the four identified compounds, both cyanidin and peonidin derivatives show interesting potentiality based on the structure-activity studies performed by Kahkonen and Heinonen [52]. In particular, authors performed a deep analysis of the main anthocyanidins and their glycosylated forms. Based on these data on the DPPH assay, the effects of hydroxylation and methoxylation at the level of B ring have a remarkable effect on antioxidant activity [53,54,55,56,57]. Cyanidin, with the presence of O-diphenyl structure in the B ring, shows high efficiency, and the presence of sugar in the 3 and 5 position slightly decreases this potentiality. Peonidin, having a methoxy group in the 3’-position, in addition to a hydroxy group in the 4’-position, shows strong activity, comparable with cyanidin derivatives. Based on these observations, and on the abundance of cyanidin-3,5-*O*-diglucoside (cyanin) and peonidin-3,5-*O*-diglucoside (peonin) in the tested EAC, these two compounds are, probably, the main responsible for the results obtained in the antioxidant assays. The presence of sugars, on the other hand, are probably responsible of the negative results obtained in the ferrozine assay, where the presence of OH and carbonyl group in the A and C ring are among the main responsible of this activity [50,51,52,53,54]. The different antioxidant assays performed shed some light on the potentiality of the EAC, but they are influenced by several different factors, such as the pH and the polarity. To confirm these observations on biological samples, we analyzed its cytoprotective potential on isolated mononuclear cells and against angiogenesis. The cytoprotective effects of the compounds present in the enriched fraction appears evident in the mononuclear cells treated with a strong oxidant (t-BOOH). This is a toxic water-soluble hydroperoxide that fairly quickly penetrates the biological membranes and interacts with cellular components with different schemes of reaction, based on the interaction between heme-containing proteins and enzymes with organic hydroperoxides, including a peroxidase mechanism and homolytic cleavage of hydroperoxide. The exposure to 50.0, 25.0, and 10 μg/mL of EAC was able to almost completely eliminate t-BOOH-increased ROS production, reaching up over 90% of ROS decrease within the cells in comparison with t-BOOH-treated ones. The EAC also showed strong activity in the angiogenesis assay. Indeed, a remarkable decrease of the CAM vessel growth in the concentration range of 3.125–25 μg/egg was observed, reaching with 6.25 μg/egg treatment results are comparable with the well-known angiostatic agent (retinoic acid). Angiostatic agents may have important clinical applications due to the occurrence of angiogenesis in several diseases (such as tumorigenesis, psoriasis, chronic inflammatory disorders, and ocular neovascularization). Matsunaga et al. [58,59] described the effects of several anthocyanins of bilberry on angiogenesis and found a direct correlation with the antioxidant effects and, in part, with the cell proliferation and migration decrease through inhibition of both extracellular signal-regulated protein kinases 1/2 (p-ERK 1/2) and protein kinase B (p-Akt). Taking into account the remarkable results obtained in the antioxidant assays performed in our study, it is not surprising to also find remarkable activity in the CAM assay.

## 4. Materials and Methods 

### 4.1. Chemicals

The 2,2-diphenyl-1-picrylhydrazyl (DPPH), potassium peroxydisulfate, 2,2′-azino-bis (3-ethylbenzothiazoline-6-sulfonic acid) diammonium salt (ABTS^•+^), 2,4,6-Tris(2-pyridyl)-S-triazine (TPTZ), ethylenediaminetetraacetic acid (EDTA), 2′,7′-dichlorodihydrofluorescein diacetate, iron sulfate heptahydrate, ferrozine, potassium peroxydisulfate, sodium phosphate dibasic, potassium phosphate monobasic, sodium acetate, iron(III) chloride hexahydrate, iron(II) chloride tetrahydrate were purchased from Sigma-Aldrich (Milan, Italy). Methanol and acetic acid were LC-MS-grade and were purchased from Merck (Darmstadt, Germany). All other chemicals and solvents used were of analytical grade.

### 4.2. Preparation of Enriched Fraction of Acidified Methanolic Extract of Callistemon citrinus Flowers (EAC)

Flowers of *C. citrinus* were collected from local nurseries in Messina (Italy), hair dried until they reached moisture lower than 2%, powdered with a mortar, and used for the extraction of anthocyanins. A total of 1.0 g of powder was extracted with acetic acid:methanol:water (1:70:29, *v*/*v*/*v*) mixture 1:10 (*w*/*v*), several folds, until the flowers were exhaustively extracted from their content in anthocyanins. The obtained volume was concentrated to 5.0 mL with a rotary evaporator and further extracted and concentrated, following by a SPE extraction by a Supelclean™ LC-18 SPE cartridge. (Supelco Ltd., Bellefonte, PA, USA). The final elution has been performed with acetic acid:methanol:water (1:70:29, *v*/*v*/*v*) mixture. The obtained anthocyanins-rich fraction was bring to dryness with a yield of ~23%. The obtained powder was stored in the dark at 4 °C. Aliquots of the powder were diluted with the above acetic acid:methanol:water mixture at the final concentration of 1.0 mg/ml (stock solution), used for subsequent analyses, and indicated as EAC.

### 4.3. Anthocyanin Profile Characterization by RP-HPLC-DAD-ESI-MS/MS Analysis

The qualitative and quantitative determination of anthocyanins was carried out using an Agilent high performance liquid chromatography system (1100 series), equipped with a photodiode-array detector (DAD) (G1315) and an ion trap mass spectrometer (6320) fitted with an ESI source operating in positive ionization mode. The chromatographic separation was carried out using a Luna Omega PS C18 column (150 × 2.1 mm, 5 µm; Phenomenex) with solvent A (formic acid 0.1%) and solvent B (acetonitrile), according to the following gradient elution program: 0–3 min, 0% B; 3–9 min, 3% B; 9–24 min, 12% B; 24–30 min, 20% B; 30–33 min, 20% B; 33–43 min, 30% B; 43–63 min, 50% B; 63–66 min, 50% B; 66–76 min, 60% B; 76–81 min, 60% B; 81–86 min, 0% B, and equilibrated 4 min for a total run time of 90 min. The flow rate, injection volume and column temperature were 0.4 ml/min, 5 µL and 25 °C, respectively. UV-Vis spectra of anthocyanins were recorded from 190 to 600 nm and chromatograms were acquired at 260, 292, 330, 370, and 520 nm to detect all polyphenols classes. Nitrogen was used as dry gas with a flow rate of 9 L/min and a pressure of 40 psi. Dry temperature was set at 350 °C. Using helium as the collision gas (1.46 × 10^−5^ bar), collision-induced dissociation spectra were obtained with a fragmentation amplitude of 1.0 V (MS/MS). The peak’s identity was confirmed by comparing retention times, UV-Vis, and mass spectra with those reported in literature. Quantification, due to the presence of poli-glycosylated and polymeric anthocyanins, were expressed as cyanidin-3-*O*-glucoside equivalents/100 g of dry extract (DE) by using an external calibration curve of the reference standard.

### 4.4. 2,2-Diphenyl-1-picrylhydrazyl (DPPH) Assay

The DPPH free radical scavenging activity was evaluated according to Barreca et al. [60] with few modifications. Briefly, freshly DPPH methanol solution (80 μM) was mixed with 20 μL of EAC (appropriately diluted with solvent) to reach the final concentration of 0.5, 1.0, 2.5, 5.0, 10.0, and 15.0 μg/mL and mixed for 10 s at room temperature (RT). The decrease in absorption at 517 nm, against blank, was measured after 30 min with a Varian Cary 50 UV-Vis spectrophotometer. DPPH^•^ concentration in the cuvette has been chosen to give absorbance values less than 1.0. The inhibition (%) of radical scavenging activity was calculated by the following equation:

(1) $$\mathrm{Inhibition}\left(\%\right)=\frac{A_{O}-A_{S}}{A_{O}}\times 100$$

where A_0_ is the absorbance of the control and A_S_ is absorbance of the sample after 20 min incubation. The half-maximal inhibitory concentration (IC_50_) is the concentration of the enriched fraction able to scavenge 50% of the radical present in the solution.

### 4.5. Trolox Equivalent Antioxidant Capacity (TEAC) Assay 

The antioxidant activity against ABTS^•+^ radical was carried out according to Smeriglio et al. [61] with few modifications. A stable stock solution of ABTS^•+^ was produced by reacting a 7 mM aqueous solution of ABTS (final concentration) with 2.45 mM ammonium persulfate (final concentration) and allowing the mixture to stand in the dark at room temperature (RT) for 12–16 h before use. At the beginning of the analysis day, an ABTS^•+^ working solution was obtained by diluting the stock solution, with phosphate buffer (pH 7.4), to an absorbance of 0.70 ± 0.02 at 734 nm. A total of 20 µL of sample solution (appropriately diluted with solvent) was added to 1.0 ml of reaction mixture to reach the final concentration of 0.5, 1.0, 2.5, 5.0, 10.0, and 15.0 μg/ml, mixed and incubated in the dark at RT for 6 min. After the incubation time, the absorbance was recorded at 734 nm using a Varian Cary 50 UV-Vis spectrophotometer, and results were expressed as IC_50_.

### 4.6. Oxygen Radical Absorbance Capacity (ORAC) Assay

The ORAC assay was carried out according to Smeriglio et al. [62]. Twenty microliters of extract solution (0.15–1.2 μg/mL) diluted in 75 mM phosphate buffer (pH 7.4), were added to 120 µL of 117 nM fluorescein solution, and incubated 15 min at 37 °C. After that, 60 µL of fresh AAPH solution (40 mM) was added to start the reaction. The fluorescence was monitored every 30 s for 90 min (λex: 485; λem: 520) using a fluorescence plate reader (Fluostar Omega, BMG Labtech, Ortenberg, Germany). Phosphate buffer and trolox (10–100 μM) were used as negative and positive control, respectively. Results were expressed as inhibition (%) of the AAPH radical activity calculating the IC_50_ values.

### 4.7. β-Carotene Bleaching Assay

The β-carotene bleaching assay was carried out according to Smeriglio et al. [60]. Three hundred and twenty microliters of different concentration of extract solution (2.5–20 µg/mL) were added to 8 mL of β-carotene emulsion. An emulsion with β-carotene, containing only vehicle, was used as negative control, whereas an emulsion without β-carotene was used to check the correct emulsion formulation. The absorbance of samples were recorded at the starting time (*t* = 0) at 470 nm using a UV-Vis Spectrophotometer (Shimadzu UV-1601, Kyoto, Japan), followed by a reading every 20 min for 120 min. During experiment, samples were incubated at 50 °C in a water bath in order to induce the β-carotene bleaching. Butylated hydroxytoluene (BHT) was used as reference compound and results were expressed as inhibition (%) of the β-carotene bleaching, calculating the IC_50_ values.

### 4.8. Ferric Reducing Antioxidant Power (FRAP)

The free-radical scavenging capacity against TPTZ radical was performed according to Smeriglio et al. [59]. The fresh-working FRAP reagent was prepared daily by mixing 25 mL of acetate buffer (300 mM, pH 3.6), 2.5 mL of 2,4,6-Tris(2-pyridyl)-S-triazina (TPTZ) solution (10 mM in 40 mM HCl), and 2.5 mL of FeCl_3_ × 6H_2_O solution (20 mM). Twenty-five microliters of sample solution (diluted 1:5 *v*/*v* with appropriate solvent) was added to 1.5 mL of daily fresh FRAP reagent pre-warmed at 37 °C, and the absorbance recorded at 593 nm, by an UV–Vis Spectrophotometer (Shimadzu UV-1601), after an incubation time of 4 min at 20 °C, using the FRAP reagent as blank. Trolox (1–100 μM) was chosen as a standard antioxidant and extract activity was expressed as micromolar trolox equivalents (μM TE).

### 4.9. Chelating Capacity on Fe^2+^

Fe^2+^ chelating capacity was evaluated as described by Smeriglio et al. [59] with some modifications. Briefly, 25 µL of FeCl_2_∙4H_2_O solution (1.8 mM) were added to 50 µL of sample solution (appropriately diluted with solvent) to reach the final concentration of 200.0, 175.0, 150.0, 125.0, 100.0, 750.0, 50.0, 25.0, 10.0, 5.0, 2.5, and 1.25 μg/mL and incubated at room temperature for 5 min. Following, 50 μL of a ferrozine solution (4 mM) was added to the reaction mixture, and the sample volume diluted to 1.5 ml with deionized water. After that, the mixture was mixed and incubated for 10 min at RT. The absorbance was read at 562 nm using an UV-Vis Spectrophotometer (Shimadzu UV-1601). The inhibition (%) of Fe^2+^-chelating capacity was calculated using Equation (1).

### 4.10. Evaluation of Intracellular ROS Production by Fluorescence Microscopy

Intracellular ROS, induced by t-BOOH treatment, was analyzed by ZOE Fluorescent Cell Imager (BioRad, Hercules, CA, USA) utilizing the 2′,7′-dichlorodihydrofluorescein diacetate dye. Human mononuclear cells (HMNC) were bought from Sigma-Aldrich and processed following the details supplied by the seller. During the cytotoxicity assay, cells were incubated in complete medium without or with 200.0, 175.0, 150.0, 125.0, 100.0, 750.0, 50.0, 25.0, 10.0, 5.0, 2.5, and 1.25 μg/mL of EAC for 24 h. For the cytoprotective assay, during the experimentation, human mononuclear cells were incubated with t-BOOH (50 μM) for 2 h in presence/absence of different concentrations of EAC (50, 25, 10, 5, and 2.5 μg/mL, final concentration). The enriched fraction was added to the culture medium 30 min prior to t-BOOH treatment. Then, the cells were washed twice with phosphate buffered saline (PBS) pH 7.4 and incubated with 2′,7′-dichlorodihydrofluorescein diacetate for 20 min at 37 °C. Blank samples, without t-BOOH and EAC, have also been prepared in each test. EAC, at the concentration used in the experiments, did not show any evidence of interference with the assays performed. After the incubation time, the cells were washed twice with PBS, and fluorescence images were acquired with ZOE Fluorescent Cell Imager, and digitalized. Digitized images were processed, by quantitative point of view, with ImageJ software version 1.8.0_112, which included background subtraction, contrast enhancement, dye front baseline correction, and signal to noise enhancement. The ROS formation has been expressed as the maximum amount of radicals obtained in the samples treated with t-BOOH.

### 4.11. Chick Chorioallantoic Membrane (CAM) Assay

The evaluation of the anti-angiogenic effect was carried out by the CAM assay on fertilized eggs of *Gallus gallus*, according to the method reported by Certo et al. [63]. Extract stock solution (50 mg/mL dissolved in ethanol) was diluted in Tris buffer (pH 7.4) in order to obtain the final concentrations (3.125–25 μg/egg), which were applied directly on the CAM surfaces. Retinoic acid (1.0 μg/egg) was used as positive control, whereas CAM treated only with Tris buffer (pH 7.4) was used as negative control. After treatments, the eggs were incubated for 24 h at 37 °C. The anti-angiogenic activity of the extract under study was evaluated by inspection of the number of blood vessel branch points in a standardized area by a stereomicroscope (SMZ-171 Series, Motic, HongKong, China) equipped with a digital camera (Moticam^®^ 5 plus). The acquired images were analyzed by the GNU Image Manipulation Program (GIMP version 2.10.2) and results were expressed both as angiogenic activity percentage (%) with respect to the negative control and as IC_50_.

### 4.12. Statistical Analysis

Data are expressed as means ± standard deviation (S.D.) or with the respective confident limits at 95% (C.L.). Statistical analyses were performed by one-way analysis of variance (ANOVA). The significance of the difference from the respective controls for each experimental test condition was assayed by using Tukey’s test for each paired experiment. A *P* < 0.05 was considered statistically significant.

## 5. Conclusions

This is the first study dealing with a deep analysis of the anthocyanin profiles of *Callistemon citrinus* flowers, which gave us the possibility to identify, for the first time, the presence of four main anthocyanins by RP-HPLC-DAD-ESI-MS/MS, obtained in remarkable amounts and by simple extraction and concentration procedures. The analysis of the antioxidant, cytoprotective, and anti-angiogenic properties show very promising activity, with results comparable to the reference compounds commonly and commercially used. Moreover, the good amounts, and the purity, along with the health promoting properties highlighted in this study, support the possibility of using the flowers of *Callistemon citrinus* as a rich and inexpensive natural source of anthocyanins.

## Figures and Tables

**Table 1:** Anthocyanin profile of EAC by RP-HPLC-DAD-ESI-MS/MS analysis. Results represent the mean ± SD (*n* = 3) of three independent experiments, and are expressed as mg cyanidin-3-*O*-glucoside equivalents (CyG/100 g of dry extract (DE)).

Peak	Compound	Rt (min)	Λmax (nm)	MS (*m*/*z*) [M + H]^+^	MS/MS (*m*/*z*) [M + H]^+^	mg CyG/100 g DE
**1**	Cyanidin 3,5-*O*-diglucoside	17.76	512	611	449, 287	295.58 ± 2.23
**2**	Peonidin-3,5-*O*-diglucoside	20.28	514	625	463, 301	182.03 ± 1.90
**3**	Cyanidin-3-*O*-glucoside	22.52	512	449	287	38.07 ± 0.54
**4**	Cyanidin-coumaroylglucoside-pyruvic acid	25.21	506	661, 595	482	13.94 ± 0.27
